# Effectiveness of various cleaning and disinfectant products on *Clostridium difficile* spores of PCR ribotypes 010, 014 and 027

**DOI:** 10.1186/s13756-017-0210-3

**Published:** 2017-06-03

**Authors:** N. Kenters, E. G. W. Huijskens, S. C. J. de Wit, I. G. J. M. Sanders, J. van Rosmalen, E. J. Kuijper, A. Voss

**Affiliations:** 1 Department of Infection Prevention and Control, Albert Schweitzer Hospital, Dordrecht, The Netherlands; 2 Department of Medical Microbiology, Albert Schweitzer Hospital, Dordrecht, The Netherlands; 3 Department of Biostatistics, Erasmus MC, Rotterdam, The Netherlands; 4 Section Experimental Bacteriology, Department of Medical Microbiology, Leiden University Medical Center, Leiden, The Netherlands; 5 Department of Medical Microbiology, Radboud University Medical Centre, Nijmegen, The Netherlands; 6 Department of Medical Microbiology, Canisius-Wilhelmina Hospital, Nijmegen, The Netherlands

**Keywords:** Cleaning/disinfecting wipes, Cleaning/disinfecting sprays, *C. difficile*, ATP, CFU

## Abstract

**Background:**

In healthcare facilities, *Clostridium difficile* infections spread by transmission of bacterial spores. Appropriate sporicidal disinfectants are needed to prevent development of clusters and outbreaks. In this study different cleaning/disinfecting wipes and sprays were tested for their efficacy against spores of distinctive *C. difficile* PCR ribotypes.

**Methods:**

Four different products were tested; 1) hydrogen peroxide 1.5%; 2) glucoprotamin 1.5%; 3) a mixture of ethanol, propane and N-alkyl amino propyl glycine; and 4) a mixture of didecyldimonium chloride, benzalkonium chloride, polyaminopropyl, biguanide and dimenthicone as active ingredients. Tiles were contaminated with a test solution containing a concentration of 5x10^6^CFU/ml spores of *C. difficile* strains belonging to PCR ribotypes 010, 014 or 027. The tiles were left to dry for an hour and then wiped or sprayed with one of the sprays or wipes as intended by the manufacturers. When products neutralized after 5 min, microbiological cultures and ATP measures were performed.

**Results:**

Irrespective of the disinfection method, the microbial count log_10_ reduction of *C. difficile* PCR ribotype 010 was highest, followed by the reduction of *C. difficile* 014 and *C. difficile* 027. Overall, the wipes performed better than the sprays with the same active ingredient. On average, although not significantly, a difference in relative light units (RLU) reduction between the wipes and sprays was found. The wipes had a higher RLU log_10_ reduction, but no significant difference for RLU reduction was observed between the different *C. difficile* strains (*p* = 0.16).

**Conclusion:**

*C. difficile* spores of PCR ribotypes 014 and 027 strains are more difficult to eradicate than non-toxigenic PCR ribotype 010. In general, impregnated cleaning/disinfection wipes performed better than ready-to-use sprays. Wipes with hydrogen peroxide (1.5%) showed the highest bactericidal activity.

## Background

Currently *C. difficile* is emerging worldwide in healthcare facilities [1]. The incidence of *C. difficile* infections doubled between 2001 and 2010 in the United States of America [2–5]. *C. difficile* is an important health threat associated with morbidity, mortality, and extra costs. The costs caused by *C. difficile* are estimated between $8911 and $30,049 per case [3, 5]. These costs arise due to direct healthcare costs and due to longer hospital stays. The yearly national excess hospital cost associated with hospital-onset *C. difficile* is estimated to be €4 billion for Europe, $1 billion in the United States of America and $280 million in Canada [5, 6]. Effective infection control measures are therefore greatly needed.

The hospital environment is known to be a key pathway for patients to acquire *C. difficile* infections (CDI). Spores of *C. difficile* can survive in hospitals for years [7]. New views on the transmission of *C. difficile* conclude that asymptomatic carriers can also introduce the bacteria into the hospital and may consequently play an important part in the transmission to other patients. Still, the chance of transmission from asymptomatic carriers is probably lower than from patients with a CDI [8, 9]. Guidelines to date only advise to take extra measures with CDI diagnosed patients, for example the guideline for disinfection and sterilization in healthcare facilities, 2008, of the Centers of Disease Control and Prevention (CDC).

To disinfect environments contaminated with *C. difficile*, it is generally advised to use an unbuffered 1:10 dilution of hypochlorite [10]. It is known that hypochlorite does not enhance sporulation and when used for environmental disinfection leads to a significant reduction of *C. difficile*-associated diarrhea [11]. However, hypochlorite has to be used in excessive concentrations to be effective, thereby increasing its toxic and corrosive properties. Therefore, alternative agents are needed to eradicate spores of *C. difficile*.

In the present study, four products were tested that are most commonly used as cleaning and disinfecting products in hospitals in the Netherlands. These products were tested for their efficacy against three different *C. difficile* PCR ribotypes, representing an outbreak related PCR ribotype (027), an endemic PCR ribotype (014) and a non-toxigenic PCR ribotype (010).

## Methods

### Compounds tested (Table 1)

Compounds A and D are both cationic surfactants, which cause membrane damage involving phospholipid bilayers of the cytoplasmic membrane [12]. Compound B, with a sporicidal claim, has hydrogen peroxide as active ingredient. Hydrogen peroxide kills bacteria by oxidizing their cell walls, stealing electrons and disrupting their chemical structures [13]. Hydrogen peroxide wipes are suggested to be sporicidal and can be used near patients for enhanced cleaning and disinfection. Compound C is classified as an alcohol, which denaturizes the proteins rapidly and causes membrane damage, which then interferes with the metabolism and causes cell lysis [12]. Alcohol is known to be non-sporicidal [12].

**Table 1:** Disinfecting cleaning wipe and spray ingredients

Wipe/Spray	Composition^a^	Product	Sporicidal claim
Wipe and spray A	Glucoprotamin 26 g/100 g (1.5%)	Incidin plus wipes	No
Wipe and spray B	Hydrogen peroxide (Hispeed H2O2™): 15 mg/g (CAS 77–22-841)	Aseptix Sterimax Sporicide wipes	Yes
Wipe and spray C	Ethanol 140 mg/g, Propane-2-ol 100 mg/g; Propane-1-ol 60 mg/g, Nalkyl amino propyl glycine (CAS 1397 34–65-9) 5 mg/g	Bacillol 30 tissues	No
Spray D	Didecyldimonium Chloride, Benzalkonium Chloride, Polyaminopropyl, Biguanide, Dimenthicone	Formula 429 spray	Not known

^a^Active ingredient

### Disinfecting cleaning wipes and sprays

Compounds A, B and C were tested in the form of a wipe, as well as in the form of a spray. Compound D presently only exists as a spray. All of the sprays and wipes combine cleaning and disinfection properties. Wipes and sprays A-C are presently used in healthcare facilities around Europe, but spray D is not used in healthcare facilities (Table 1). Wipes A and C are ready to use, whereas wipe B needs to be prepared according to the manufacturer’s instructions.

### Bacterial strains

*C. difficile* strains 010, 014 and 027 were obtained from the Dutch National Reference Laboratory (Type 010 Leeds/Leiden collection; Type 014 Brazier ATCC 43600 and Type 027 Brazier R20291). The strains were grown 48 h at 37 °C on a Brazier agar in an anaerobic chamber. Brazier medium was chosen since it has spore-germinating properties [14].

### Efficacy of cleaning/disinfectant products to *C. difficile* spores

After culturing, the bacterial strains were suspended in phosphate buffered saline (PBS) and adjusted to a McFarland standard of 0.5. Vegetative cells were killed by heating for 20 min at 65 °C in a water bath [15]. To mimic low and high organic contamination in hospital environments, spores were suspended in two different test solutions; A and B. Test solutions contained 3% bovine serum albumin with 0.3% sheep erythrocytes (A) or 12% bovine serum albumin with 10% sheep erythrocytes (B), to mimic ‘low’ and ‘high’ organic contamination conditions respectively, similarly as applied in a study by Diab-Elschahawi et al. [16]. All tests were performed in triplicate. As a positive control three tests were carried out with the inoculum, but without the decontamination step. For the negative control sterile water was used instead of the inoculum and no decontamination step was applied.

Standardized ceramic tiles (Villeroy & Boch, Art.Nr:3709/PA00) measuring 5x5cm were used as test surface [16]. Tiles were sterilized for 15 min at 121 °C. The tiles were contaminated with 0.1 ml of test suspension corresponding with 5x10^6^CFU/ml spores. The test suspension was spread with a spatula and dried in the laminar airflow cabinet for one hour.

A reduction of spores does not necessarily mean that all spores are killed; it is possible that only growth is inhibited. Therefore we chose to use two different methods to test efficiency: adenosine triphosphate (ATP) to measure reduction of *C. difficile* and counting colony forming units (CFUs) to measure killing of *C. difficile* spores. ATP was measured before and after tiles underwent cleaning/disinfection with a wipe or spray. Clean trace 3 M swabs were used according to the manufacturer’s instruction and relative light units (RLU) were measured in a clean trace NG 3 M luminometer [17]. To measure CFUs, tiles were wiped with a cloth or sprayed and then wiped with a paper towel. A standardized sweeping technique was used; wiping was performed starting in the left upper corner performing a meander-like pattern, with 4 turns, ending in the right lower corner. Tiles were then placed into neutralizer, consisting of lecithin 3 g/l, L-histidine 1 g/l, saponin 30 g/l in diluent (tryptone, pancreatic digest of casein 1.0 g/l, sodium chloride 8.5 g/l). After 2 min in the neutralizer (10 ml) and 3 min of horizontal shaking (150 rpm) with glass beads (15 g; 5 mm) an aliquot of the suspension (0.1 ml) was plated on Brazier’s agar. CFUs were counted after 48 h of incubation at 37 °C on Brazier’s agar in an anaerobic chamber.

### Statistical analysis

The log reduction was defined as the logarithm with base 10 of the relative reduction of CFUs and RLUs. Log reductions were summarized using means and standard deviations. Analysis of variance (ANOVA) was used with the log reduction of CFUs and RLUs as dependent variable and bacteria (*C. difficile* strains belonging to PCR ribotypes 010, 014, 027), wipes A-C and sprays A–D, and level of pollution (3% and 12%) as independent variables. Two-way interaction effects of bacteria, spray/wipe, and level of pollution were included in the model when statistically significant. The results of the ANOVA are summarized using the estimated marginal means, which are the predicted values of the dependent variable (log reduction of CFUs/RLUs) adjusted for the effects of covariates. Tukey’s multiple comparisons of means were used to assess the differences between the different products and bacteria. All statistical analyses were carried out in R version 3.1.1. (Vienna, Austria) [18], and a two-sided significance level of 0.05 was used.

## Results

### CFU - reduction

The overall CFU reduction was highest for *C. difficile* PCR ribotype 010 (log_10_ 4.50, 95% CI 4.37–4.69), followed by the effect against *C. difficile* PCR ribotype 027 (log_10_ 3.60, 95% CI 3.44–3.76) and *C. difficile* PCR ribotype 014 (log_10_ 3.75, 95% CI 3.59–3.92). The CFU reduction of the tested products was significantly less for *C. difficile* 014 and 027 in comparison to *C. difficile* 010 (*p* < 0.001). The marginal estimated mean in log_10_ CFU reduction per product is shown in Table 2.

**Table 2:** Mean log_10_ bacterial load reduction and mean log_10_ RLU reduction of cleaning/ disinfection products with 95% confidence intervals (CIs)

Product	Log_10_ CFU reduction	Log_10_ RLU reduction
Wipe A	3.91 (CI 3.66–4.16)	1.86 (CI 1.78–1.94)
Spray A	3.08 (CI 2.83–3.33)	1.67 (CI 1.67–1.84)
Wipe B	5.29 (CI 5.04–5.54)	1.86 (CI 1.77–1.94)
Spray B	4.08 (CI 3.83–4.33)	1.45 (CI 1.36–1.53)
Wipe C	4.69 (CI 4.44–4.94)	1.67 (CI 1.58–1.75)
Spray C	3.09 (CI 2.84–3.34)	1.54 (CI 1.46–1.62)
Spray D	3.59 (CI 3.34–3.84)	1.56 (CI 1.48–1.64)

Wipe B had the highest log_10_ CFU reduction of 5.29 (95% CI 5.04–5.54) for the wipes and spray B had the highest log_10_ CFU reduction of 4.08 (95% CI 3.83–4.33) for the sprays. The efficacy between wipe B and spray B is significantly different (*p* < 0.001). Wipe B with a 1.5% hydrogen peroxide concentration was the only product to reach a 5log_10_ CFU reduction for all the *C. difficile* strains tested. Overall the wipes were more effective than the sprays with the same active ingredient (all; *p <* 0.001).

In the experiment no significant difference in efficacy of the tested products between test solution A and B (*p* = 0.50) was found according to ANOVA.

**Fig. 1:**
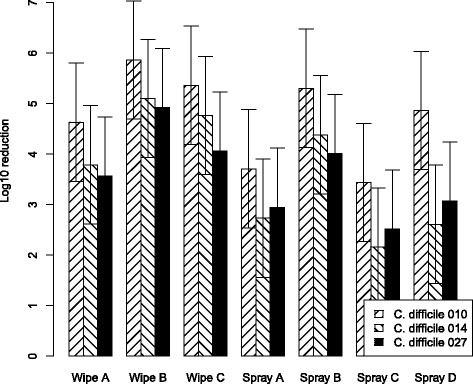
CFU reduction with “low” organic contamination (solution A) Mean log_10_ bacterial removal from tiles examining efficacy of disinfecting cleaning wipes and spray with a 3% test soil against 5x10^6^CFU/ml of *C. difficile* PCR ribotypes 010, 014 and 027. Data are the estimated marginal mean of 3 triplicates, and bars represent 95% prediction intervals

**Fig. 2:**
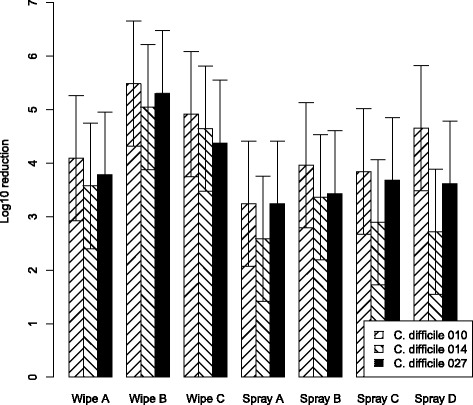
CFU reduction with “high” organic contamination (solution B) Mean log_10_ bacterial removal from tiles examining efficacy of disinfecting cleaning wipes and spray with a 12% test soil against 5x10^6^CFU/ml of *C. difficile* PCR ribotypes 010, 014 and 027. Data are the estimated marginal mean of 3 triplicates, and bars represent 95% prediction intervals

The estimated marginal mean log_10_ CFU reductions of *C. difficile* for all products and test solutions are shown in Figs. 1 and 2. Figure 1 demonstrates that wipes B and spray B were the most effective against all tested PCR ribotypes. A discrepancy in mean log_10_ bacterial removal for wipe B is seen between the two organic contamination solutions, as wipe B performed better with “high” organic contamination (B). Figure 2 also shows that in a high organic contamination environment, wipe B was especially most effective for ribotype 027.

### ATP – reduction

On average the RLU log_10_ reduction of *C. difficile* 010 was 1.70 (95% CI 1.65–1.76), for *C. difficile* 014 1.67 (95% CI 1.61–1.72) and for *C. difficile* 027 1.64 (95% CI 1.58–1.69).

The tested products had different RLU log_10_ reductions (Table 2). The most effective products in removing the test soil were wipes A and B with a RLU log_10_ reduction of 1.86 (95% CI 1.77–1.94). For products A and C no significant difference was found in effectiveness between the spray and wipe (*p* = 0.62 and *p* = 0.36). Product B had a significant difference in its effectiveness between the wipe and spray (*p* < 0.001).

The RLU log_10_ reduction differed significantly between test solutions A and B (*p* < 0.001), whereas no significant difference for RLU reduction was seen between the different *C. difficile* strains (*p* = 0.16).

**Fig. 3:**
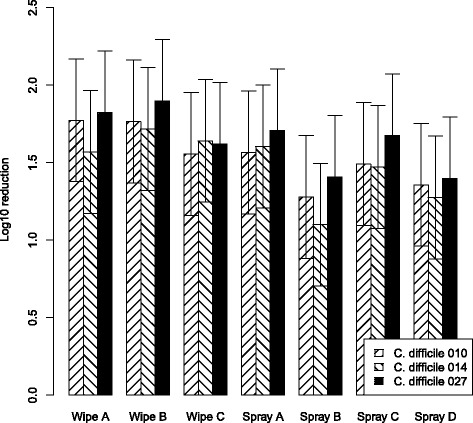
RLU reduction with “low” organic contamination (solution A) Mean log_10_ ATP reduction from tiles examining efficacy of disinfecting-cleaning wipes and spray with a 3% test soil against 5x10^6^ CFU/ml of *C. difficile* PCR ribotypes 010, 014 and 027. Data are the estimated marginal mean of 3 triplicates, and bars represent 95% prediction intervals

**Fig. 4:**
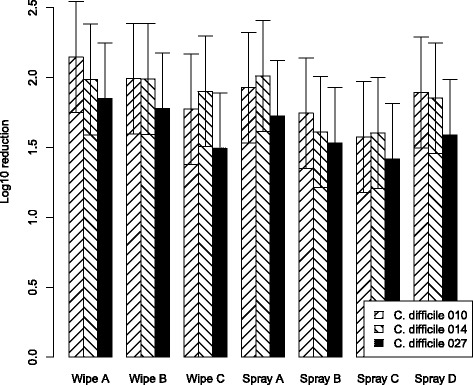
RLU reduction with “high” organic contamination (solution B) Mean log_10_ ATP reduction from tiles examining efficacy of disinfecting-cleaning wipes and spray with a 12% test soil against 5x10^6^CFU/ml of *C. difficile* PCR ribotypes 010, 014 and 027. Data are the estimated marginal mean of 3 triplicates, and bars represent 95% prediction intervals

The estimated marginal mean of RLU log_10_ reduction per bacteria for both test solutions is shown in Figs. 3 and 4. Figure 3 reveals that all wipes (except wipe C) and sprays had greatest protein removal against ribotype 027. In addition a discrepancy in protein reduction for *C. difficile* PCR ribotype 027 between the organic solutions A and B is shown, the activity of the wipes and sprays was smaller in the ‘high’ organic solution.

## Discussion

CDI is a serious infection, with an all cause 30-day mortality of 15% or greater, that warrants a variety of infection control measures to prevent and control its occurrence [19]. Effective cleaning and disinfection is an essential prerequisite to prevent the spread of CDI within healthcare settings. Presently, chlorine-based products are the mainstay with regard to environmental disinfection in the Netherlands, but alternative, ready-to-use products are needed to ensure consistent cleaning. We therefore tested the effectiveness of different cleaning/disinfecting wipes and sprays against spores of *C. difficile* PCR ribotypes 010, 014 and 027. These ribotypes were chosen because of their differences in virulence and transmission potential. *C. difficile* ribotype 010 does not produce toxins and therefore is unable to cause CDI in humans. In contrast, *C. difficile* PCR ribotype 027 is known for its “hypervirulence”. It is associated with increased morbidity and mortality [15, 20, 21], as well as its potential to cause large outbreaks. Currently, this type is found in 1.2% of all characterized isolates sent to the National Reference Laboratory in the Netherlands [21]. The third tested strain PCR ribotype 014 produces toxin A and B and is the most prevalent (17%) PCR ribotype in the Netherlands [21].

The overall effectiveness of products measured by log_10_ CFU reductions ranged from 3.09 (spray A) to 5.29 (wipe B). While to date a European standard for an in vivo test that mimics the real-life situation for sporicidal effectiveness is missing, the EN 13704 ‘suspension test’ requires a 3 log_10_ CFU reduction after 60 min. All products, in all application forms, would therefore pass this European norm. Given the fact that higher numbers of spores are found in the hospital environment [20] and that patients with a CDI can excrete up to 1 × 10^7^ spores per gram feces [5], a more realistic EN test should be developed that mimics real-life bacterial/spore loads and cleaning times of less than 60 min. We would recommend a significantly higher (e.g. a 5 log_10_) CFU reduction for effective control of *C. difficile* transmission, as was also proposed by Fraise et al. [20]. Our tests show that this requirement is feasible, as shown by the fact that wipe B achieved a 5.29 log_10_ CFU reduction.

When comparing the mean log_10_ CFU reductions by application type (wipe versus spray), it became obvious that the ready-to-use wipes were outperforming the sprays using a paper towel by 0.81 to 1.60 log_10_ CFU reductions. The differences in log_10_ CFU reduction between the wipe and spray with the same active ingredient were consistently observed for all products tested in both application forms (A, B and C). While not as pronounced, the differences in log_10_ CFU reductions were also apparent in log_10_ RLU reductions, with the three highest log_10_ RLU reductions seen for wipes and the lowest for sprays. This difference between wipes and sprays could possibly be explained by the “mechanical” effect involved with cleaning/disinfecting. Studies similar to ours, but using detergent wipes achieved an average log_10_ CFU reduction of 1.63, which is exactly within the range of difference we observed with wipes and sprays [22, 23]. Clearly, the application form is responsible for a significant part of the effect in addition or combination with the disinfecting active compound. As we compared wipes against sprays plus paper towels, some may argue that the difference in effect is due to the difference in mechanical effect of the different materials used for wiping. Based on a study by Diab-Elschahawi et al., who compared microfibers, cotton cloths, sponge cloths and paper towels for their decontamination abilities, without finding a significant difference [16], we conclude that the difference between wipes and sprays in our study cannot be explained by the difference in wiping material.

Although sprays were used according to the suppliers’ instructions, surface coverage as well as the actual contact time and number of wiping movements might be different to the use of impregnated wipes. Wipes B and C were available as ready-to-use wipes and wipe A needed to be prepared in a reusable container. Ready-to-use wipes eliminate the possibility of human errors that could make the disinfectant less effective or make the wipes unnecessarily toxic.

In addition to the application method and the compound used, our results indicate that the individual *C. difficile* strain is of importance with regard to the effect of cleaners/disinfectants. While CFU reductions were highest for the non-toxin producing *C. difficile* ribotype 010 in a low organic contamination environment, they were lower for the clinically more important ribotypes 014 and 027. Interestingly, the differences in effectiveness were less pronounced and, in the case of wipe B, even reversed in a high organic contamination environment. While our results in this regard are not fully conclusive, they certainly indicate the importance of including a variety of clinically relevant ribotypes when evaluating the effect of disinfectants against *C. difficile*.

## Conclusion

In conclusion *C. difficile* spores of 014 and 027 strain are harder to eliminate compared to those of the non-toxigenic strain 010. Future studies should use these more resilient types of *C. difficile* to ensure the needed in-vivo effect. Impregnated cleaning/disinfection wipes generally outperform ready-to-use sprays, even if based on the same active ingredient, and should thus be preferred over sprays for the daily cleaning/disinfection in rooms of patients with CDI. While all products pass current EN 13704 standards we believe that - given the in-vivo load of Clostridium spores - higher standards should be set, such as those achieved by the products based on 1.5% of hydrogen peroxide.

## Abbreviations

ANOVA: Analysis of variance
ATP: Adenosine triphosphate
CDI: *C. difficile* infections
CFUs: Colony forming units
PBS: Phosphate buffered saline
RLU: Relative light units
